# Protection of Vaccine Preventable Diseases in a Population of HIV-Infected Children: A 3 Years Prospective Study

**DOI:** 10.3390/vaccines9111331

**Published:** 2021-11-15

**Authors:** Eugenia Bruzzese, Federica Pagano, Alfredo Diana, Liana Punzi, Alfredo Guarino

**Affiliations:** Department of Translational Medical Sciences, Section of Pediatrics, University Federico II of Naples, 80100 Naples, Italy; paganofederica1405@gmail.com (F.P.); alfredodiana93@gmail.com (A.D.); punziliana@gmail.com (L.P.); alfguari@unina.it (A.G.)

**Keywords:** HIV infection, vaccines, immune response, children

## Abstract

Human Immunodeficiency Virus (HIV) infected children have a 30–70% chance of being incompletely immunized and may not respond serologically with the same magnitude or durability as uninfected children. The aim of the study was to describe the rate of protective antibodies titre and the persistence of the response against four recommended vaccinations in HIV infected children and adolescents. A two-phase observational study was performed in which protective IgG antibodies to measles, mumps, rubella and hepatitis B were determined and monitored for 12 and 24 months, in 26 perinatally HIV-infected children. The rate of protection for rubella and hepatitis B was significantly lower in the HIV group compared to the control group (92% vs. 65% for rubella and 78.4% vs. 45.4% for hepatitis B; *p* < 0.05). Children who received primary vaccination after initiating combination antiretroviral therapy (cART) had a higher rate of response. Seronegative patients who received a booster dose of vaccine had a good immunological response. HIV infection is associated with a lower response to vaccines against rubella and hepatitis. The beginning of cART before vaccination may be associated with a better response. The evaluation of the serological response is crucial in children with HIV infection in order to evaluate the protection of vaccine preventable diseases.

## 1. Introduction

Immunization has been identified as one of the most cost-effective interventions in global health. The incidence of measles, mumps, rubella and other vaccine-preventable diseases has dramatically decreased in countries where the vaccination is integrated into the national immunization programs, and data are available indicating that the prevalence of protective antibodies to these viruses is high in the general population [1,2]. Despite the Mumps, Measles, Rubella & Varicella (MMRV) vaccine showing an efficacy of 90% or more in preventing measles, mumps, rubella and varicella in otherwise healthy children [3], little is known about the serologic response in HIV-infected children. Several studies have shown that children born to human immunodeficiency virus (HIV)-infected women have a 30–70% chance of being incompletely immunized [4,5,6]. Low vaccination coverage may increase the morbidity and mortality risk from vaccine-preventable diseases among such children [7]. A combination antiretroviral (cART) dramatically improved survival among children with perinatally acquired HIV increasing the risk of inadequate protection to vaccine-preventable illnesses such as measles.

In addition to suboptimal vaccination coverage, perinatally HIV-infected children may not respond serologically with the same magnitude or duration as children without HIV infection. Even in children who received cART, the prevalence of protective antibody levels following primary immunization in asymptomatic or symptomatic HIV-infected children was highly variable, ranging from 38% to 42% for measles and rubella [8].

Anamnestic immune response after vaccination is a necessary requirement for long-term protection against the targeted pathogens. [9]. Children affected by HIV/AIDS are at risk of more severe illness than healthy children during common viral and bacterial infections, since the HIV infection is associated with lower lymphocyte counts; furthermore, despite B-Lymphocyte function improving following cART initiation, memory responses remain lower after achieving viral control [10,11]. Since HIV-infected children have an increased risk of severe measles disease and complications compared with HIV-unexposed children, [12] measles vaccination is safe and strongly recommended in children with HIV infection with severe immunodeficiency (e.g., CD4 *<* 200 cells/mm^3^ or <15% of total lymphocytes and physical signs of such immunodeficiency). A recent descriptive analysis of the Vaccine Adverse Event Reporting System (VAERS) database reporting vaccination-induced adverse events (AEs) among HIV-positive persons in the United States, supported the current general recommendations that for HIV-positive persons, inactivated vaccines are indicated, and live attenuated vaccines should be considered safe only in the absence of immunodeficiency [13]. The increased susceptibility to measles during early infancy in HIV-exposed infants may be explained by lower levels of maternally acquired measles antibody than HIV-unexposed [14]. Furthermore, HIV-infected, antiretroviral-naïve children have a reduced serological response to primary measles vaccination and an increased waning of immunity compared with HIV-uninfected and HIV exposed but uninfected children [15,16]. A study in HIV-infected adults in the United States reported that the prevalence of protective antibodies to measles, mumps, and rubella were 67, 91, and 95%, respectively [17]. Recently it was shown that the MMR vaccination in HIV infected adults is able to induce a seroprotective response against measles, mumps, and rubella in 96.4, 70.7, and 98.0% of patients, respectively, with no significant difference compared to that observed in uninfected controls and with no adverse effects [18]. Interestingly, although the vaccination is effective in inducing a seroprotective response in people with HIV infection, this response seems to decrease over time. The timing of cART initiation during early infancy has an on-going effect on the immune responses to booster-dose of some vaccines; it has been demonstrated that HIV-infected children not on cART had poorer memory responses at primary vaccination compared with HIV-infected children on ART; similarly, the magnitude of memory response is less robust compared to HIV-exposed-uninfected children [19]. A recent metanalysis raises further concern regarding long-term immunogenicity of a two-dose schedule given early in life, as antibody titre in HIV-infected children on ART wane over time and suggests the need for future studies on long-term waning of immunogenicity after early vaccination in HIV-infected children treated with cART [20].

Aims of this study were to describe the immunization coverage, to evaluate the rate of protective IgG titre against four recommended vaccinations, and to determine the persistence of protective response in perinatally HIV-1- infected children and adolescents followed up at the referral center of Pediatric Infectious Diseases in the Campania Region, Italy.

## 2. Materials and Methods

Our research is a two-phase observational study: in 2018 we conducted a cross-sectional study, followed by a three-year prospective cohort study.

### 2.1. Seroprevalence Study

In the first phase, we performed a cross-sectional study to compare the presence of protective IgG antibodies to four recommended vaccinations previously administered (measles, mumps, rubella, hepatitis B) in HIV-infected and uninfected children. Enrolled patients were children followed by our Pediatric Infectious Diseases reference center who came for clinical examination between January and December 2018, including HIV-positive patients, who had been formerly diagnosed with perinatal HIV infection. Controls included HIV-negative children, who were followed for other clinical conditions without any immunosuppressant effect (in most cases children with an anamnestic contact with tuberculosis but not infected, children with reactive lymphadenopathy and children with poor weight growth). All participants were tested for IgG and IgM antibodies (T0) and classified by protective/non protective IgG titre.

### 2.2. Prospective Cohort Study

In the second phase of the study, the IgG titre was repeatedly determined in HIV-positive patients during a two-year follow-up to evaluate the persistence of response.

Children who had no protective titre at the first determination received a booster dose of MMR/Hepatitis B Virus (HBV) vaccine as indicated by international guidelines [21], and then were re-tested for IgG antibodies eight to 12 weeks later.

The all HIV-positive group was tested again twice to observe the persistence or decline of protective IgG titre at:

- T12: 12 ± three months after T0
- T24: 12 ± three months after T12

### 2.3. Data Collection and Laboratory Testing

Medical records were used to collect demographic and clinical data at the time of enrollment: date of birth, sex, date of diagnosis, HIV immunological and clinical class, prior measles, mumps, rubella, and hepatitis B vaccination, anti-measles IgG antibody titer, anti-mumps IgG antibody titer, and anti-rubella IgG antibody titer, anti-HBV IgG antibody titer, type, and duration of antiretroviral therapy.

**Figure 1:**
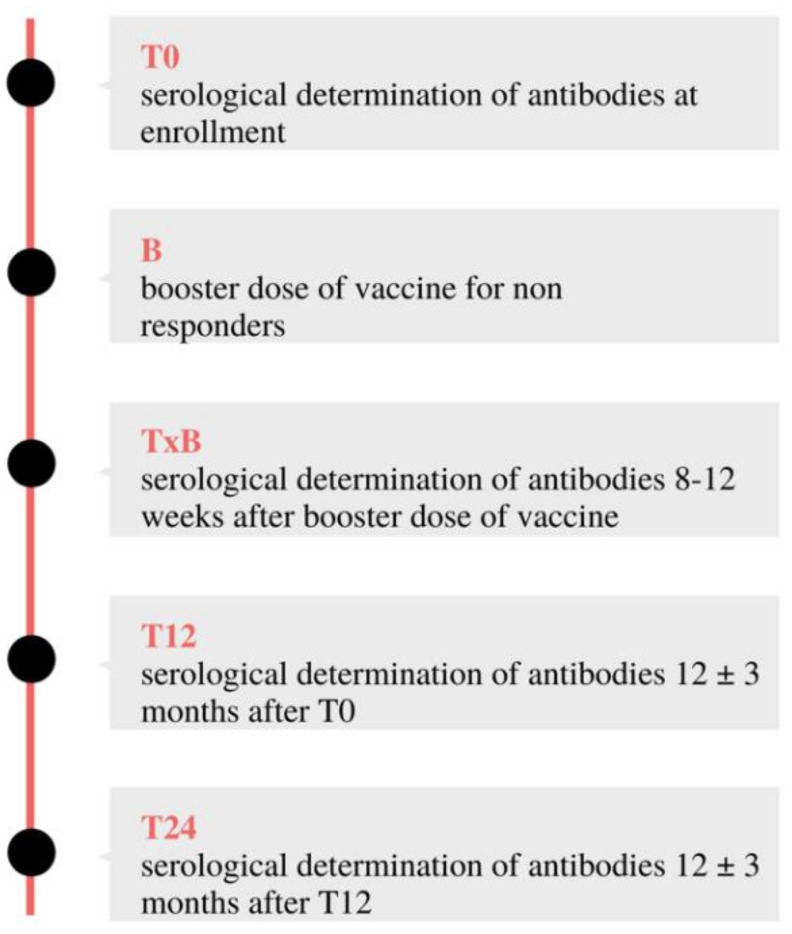
Testing and vaccine booster schedule.

Antibody levels to the four viruses were also measured at eight to 12 weeks in those who received a vaccine booster (TxB), and yearly in all participants in the second phase of the study (T12 and T24) (Figure 1). At each antibody titre determination, laboratory testing included CD4 and CD8 cell counts and plasma HIV-1 RNA, always according to the routine laboratory follow-up schedule and good clinical practice guidelines for pediatric HIV patients. All samples were processed by internal hospital laboratories. IgG antibodies titer determination was performed using chemiluminescent immunoassay (CLIA) with the following cutoff values:

- anti-measles IgG: AU/mL <13.5: Negative; 13.5–16.5: Undetermined; >16.5: Positive.
- anti-mumps IgG: AU/mL <9: Negative; 9–11: Undetermined; >11: Positive.
- anti-rubella IgG: IU/mL <7: Negative; 7–10: Undetermined; >10: Positive.
- anti-HBV IgG: mIU/mL <10: Negative; >10: Positive.

Seropositivity was defined by the presence of a positive specific IgG titre above the cut-off values. The plasma HIV-1 RNA count was based on RT-PCR with a maximum limit of detection of 40 copies.

To assess immunization coverage, defined as the proportion of subjects who received all the required vaccine doses as recommended by the Italian vaccination schedule, we retrospectively recorded the vaccination history from medical records. All patients provided written informed consent according to local procedures and to the 1975 Declaration of Helsinki. The study was reviewed and approved by the ethical committee of the University of Naples Federico II (protocol number 113/18; date of approval June 2018).

### 2.4. Sample Size and Statistical Analysis

Data are presented as mean ± SEM, median and IQR, number (%). Categorical variables were compared using the Chi-2 test and Fisher’s exact test; continuous variables were compared using the unpaired Student’s t-test and the Mann-Whitney U test. Repeated measures were analyzed using a one-way ANOVA test. The statistical analysis was conducted using IBM SPSS Statistics, release 26.0. A *p* value of less than 0.05 was considered significant.

## 3. Results

### 3.1. Population Description

Twenty-six perinatally HIV infected patients and 37 uninfected controls were enrolled. The main demographic characteristics of the enrolled children are shown in Table 1.

Clinical and immunological characteristics of HIV infected children at enrollment are shown in Table 2. None of the enrolled patients at enrollment had severe immunodeficiency (e.g., CD4 *<* 200 cells/mm^3^, or CD4 < 15%) or showed physical signs of such immunodeficiency, and all were on antiretroviral therapy. The median age at diagnosis was 14 months (range 1–151 months), the median value of CD4 at diagnosis was 371 cells/mm^3^ (range 3–2854 cells/mm^3^).

### 3.2. Seroprotection Rate in HIV Infected and Uninfected Children

A complete serological dataset on protection against the four vaccine preventable diseases were available for 26 of the 30 enrolled HIV-infected children. All HIV infected and uninfected enrolled patients had received the primary vaccination for MMR and Hepatitis B at enrollment. In the HIV group, nine patients started the vaccination program before the beginning of cART, 12 received the vaccination after the beginning of cART, and for nine patients this information was not available, as they were referred by other clinical centers and some information was lost. Looking at the rate of seroprotection for measles, mumps, rubella and hepatitis B, we found a lower response rate for all vaccines in the HIV group compared to the control group, although the statistical significance was reached only for rubella and hepatitis B (Table 3).

**Figure 2:**
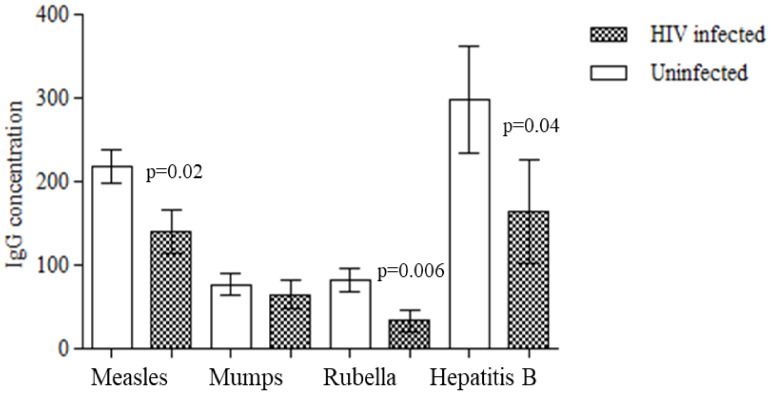
The mean serum concentration of IgG to measles rubella and hepatitis B viruses was significantly lower in children with HIV infection acquired perinatally compared to uninfected controls. No significant difference was observed in mean serum concentration of IgG for mumps between groups.

To evaluate the influence of immunological status on vaccine response, we compared the rate of seroprotective response in HIV-infected children who received primary vaccination before or after the beginning of cART. Children who received vaccines after the beginning of cART were more responsive compared to children receiving vaccines before being started on cART, although the difference was not significant (Measles 75% (9/12) vs. 44% (4/9); Mumps 75% (9/12) vs. 55% (5/9); Rubella 75% (9/12) vs. 66.6% (6/9), HBV 73% (8/11) vs. 22% (2/9), respectively). In addition, no significant difference was detected in the mean age and in the mean CD4 cell count between children with a protective IgG titre and children seronegative for antibodies to measles, mumps, rubella, and hepatitis B at enrollment. Finally, we tested the hypothesis that the magnitude of the response to vaccine was lower in HIV-infected children than uninfected children. A significantly lower concentration of IgG against measles (141.1 ± 25.98 vs. 218.1 ± 19.42 AU/mL; *p* = 0.02), rubella (33.73 ± 12.98 vs. 82.64 ± 13.86 IU/mL; *p* = 0.006) and hepatitis B (164.3 ± 62 vs. 298.5 ± 63 mIU/mL; *p* = 0.04) was detected in HIV-infected children compared to controls (Figure 2). To explore the hypothesis that the magnitude of immune response against vaccines could be influenced by the time, we divided the HIV-infected children and the controls in two groups based on age: children younger and children older than 10 years of age. The mean IgG serum concentration against measles, mumps and rubella was significantly higher in infected children younger than 10 years compared to children older than 10 years of age (209.6 ± 45.7 vs. 96.9 ± 26.9 AU/mL, *p* = 0.03; 127.1 ± 40.5 vs. 32.1 ± 9.8 Au/mL, *p* = 0.006; 69.33 ± 34.9 vs14.9 ± 4.1 IU/mL, *p* 0.04; respectively); no difference was observed between the groups for hepatitis B. In contrast, no difference for all vaccines was registered in mean serum IgG concentration between children younger or older than 10 years of age in the group of uninfected children.

### 3.3. Evaluation of Vaccine Booster Dose Effect in HIV Infected Patients

We offered a booster dose of the vaccine to the families of the children who were not protected at the first determination. Four out nine HIV infected patients without protection against measles, rubella or mumps received a single MMR booster. In the booster group the seroconversion rate was 100% for measles, 75% (3/4) for mumps and 66.6% (2/3) for rubella. Seven out 12 HIV infected children seronegative for hepatitis B received a single booster for hepatis B and three months later six out of seven patients (85.7%) showed a serum protective titre against hepatitis B. After the booster dose the rate of protective titre was 88.5% (21/26) for measles, 81% (19/26) for mumps and rubella and 73% (16/22) for hepatitis B.

### 3.4. Long Term Follow-Up of Seroprotective Rate in HIV Infected Patients

**Figure 3:**
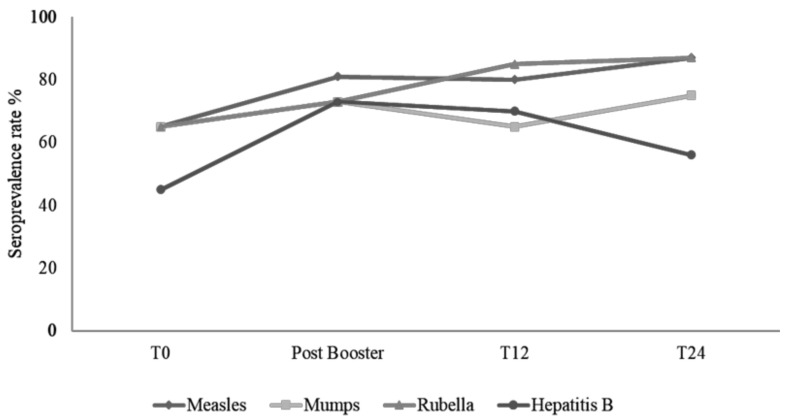
Trend of seroprotective rate against the four vaccines in HIV-infected children. The seroprotection rate increased for all vaccines after the booster dose administered to children negative at enrollment. A slight decrease is observed at T12 for measles due to one child who lost the protective antibodies after booster. A relevant reduction in the prevalence was observed for hepatitis B. The apparent increase in the seroprotective rate for mumps, rubella and measles at t 24 is due to a reduction in the number of patients still in follow-up.

During the two years of follow-up, none of the children showed significant modifications in immunological and virological parameters. No difference in the mean concentrations of CD4 cells (871.59 vs. 865.23 cells/mm^3^ respectively) and no difference in the number of children with HIV RNA < 40 copies/mL (18/21 vs. 16/16 respectively) between the T12 and T24 time points was observed. During the first 12 months, six patients were lost to follow up. One family moved to another country for work reasons, and five other patients were transferred to an infectious disease center for adults. A persistent protective response against measles, mumps, rubella and hepatitis B was observed in 16/20 (80%), 13/20 (65%), 17/20 (85%) and 12/17 (70.5%), respectively. Analyzing the data available for the 20 patients in follow up at 12 months, only one patient who received the booster for measles lost the protective antibodies titre, whereas in three patients, antibodies for primary vaccination for mumps fell below the protective threshold, and the same was observed for hepatitis B in one child. Twelve months later (T24), the sero-protective titre was determined again in 16 HIV-infected patients, as four more patients were lost to follow up. We found a protective titre in 14/16 (87%) children for measles, in 12/16 (75%) children for mumps, in 14/16 (87%) for rubella and in 9/16 (56%) for hepatitis B. Four patients who received a booster for Hepatitis B lost their protection, whereas none of the children lost protection for measles, mumps or rubella. Figure 3 shows the trend of the protection rate during the follow-up.

## 4. Discussion

In the present study we evaluated the immunization and the rate of seroprotection against four specific vaccines included in the current Italian recommendations to the pediatric population, in a population of perinatally HIV-infected and HIV-negative children. The four vaccines were selected due to the low risk of coverage in otherwise healthy children and of severe clinical outcome or complications in perinatally HIV-infected children. Although all infected children received primary vaccination, a lower prevalence of immunity was observed for all four vaccine-preventable infectious diseases in young HIV-infected patients compared to the uninfected children, which was significantly lower for rubella and hepatitis B. A low seropositivity/seroprotection rate was observed even if patients had, at time of enrollment, a well-controlled HIV infection, as judged by HIV-RNA ≤ 50 copies/mL and CD4 count ≥ 500 cells/mm^3^. This supports the evidence of a progressive decline of immunity to vaccinations in HIV-1 infected children compared with healthy individuals, even in subjects on cART. We couldn’t find any significant correlation between duration of infection or immunological status at diagnosis of HIV infection and the rate of seroprotection at enrollment. The rate of seroprotection was similar in HIV-infected children who had moderate to severe immunological impairment (immunological class 2 or 3) compared to children without immunological deficit (immunological class 1) at diagnosis.

In a study by Bekker et al., eight children with HIV infection showed protection against measles, mumps, and rubella ranging from 38 to 42%. In our population the rate of protection for the same vaccine preventable infections was close to 60%. We can explain the difference considering that the evaluation of protection rate in the study of Bekker et al. was performed in children before they started cART; in addition, cART used in our patients was probably different and more effective. In the last 10–15 years the drugs used as a first-line therapy in HIV infection in children have changed and new drugs have been approved for use in children. This may have positively influenced the immune response and therefore the ability to maintain a protective titre against vaccines for a longer time. Our population had a median value of CD4 cells equal to 371 cells/mm^3^ at diagnosis, indicating a moderate-severe immunological deficit and therefore a possible ineffective response to vaccinations. We tried to demonstrate a difference in the rate of seroprotection against the four vaccines in children with a severe-moderate immunological impairment compared to children with no immunological impairment at the time of HIV diagnosis. Even in this case, we did not find any significative differences, although the proportion of children with severe immunological impairment in the group with no response to vaccines was higher.

However, children who had no protective titre at enrollment and received a booster dose of vaccine showed a satisfactory response and were able to maintain the seroprotective titre during 24 months of follow-up in a significant proportion. Only one patient showed a reduction of antibodies against measles during the follow-up, and five children were seronegative against hepatitis B after 24 months. These findings suggest that patients with HIV infection and good clinical and immunological control of the infection are able to mount a good and specific immune response against different viral infections. In any case, despite a good initial response to primary vaccination or booster dose, the subsequent loss of detectable antibodies raises the issue of the need to monitor susceptible individuals [22]. In keeping with this observation, the mean serum IgG concentration against measles, mumps and rubella was significantly higher in younger HIV infected children compared to older children. This finding, true only in HIV infected children, supports the hypothesis that this clinical condition put children at risk for losing their protective antibody titre over time. In contrast, the drop observed for hepatitis B antibodies 24 months after the booster dose probably does not necessarily have to be linked to the HIV infection. It is well-documented that the protective titre against hepatitis B can also decrease over the time in immunocompetent children. A single booster dose of vaccine is almost always able to increase the antibody titre up to protective levels [23].

Our data confirm that serologic immunity was associated with the timing of cART initiation, especially for the HBV vaccine, with a larger proportion of children, immunized after starting cART, having seroprotection at enrollment. Previous studies had already demonstrated that early cART initiation allows better immune response to vaccines [24] and maintenance of the memory B cells [25]. This hypothesis is confirmed by Succi et al., who described an association between serologic immunity for HBV vaccine and the timing of cART initiation, with a larger proportion of children initiating cART prior to 12 months of age having seroprotection at four years of age [26].

One study has reported that a significant proportion of HIV-infected children are not initially protected against tetanus and that many who mount an initial immune response experienced waning antibody response long-term [27].

HIV-infected children are at risk of low vaccination coverage, poor immunologic response, and rapid decline of antibodies after immunization. Complete and timely vaccination of perinatally infected children is critical to effective protection from severe infections due to the impairment of immunological response and possible outbreaks of infectious diseases such as measles and rubella. A better understanding of waning immunity to HBV, measles, and rubella in HIV-infected children, as well as the need for booster doses, is needed [28]. Increasing the body of evidence on vaccine-preventable diseases in HIV-infected children is even more relevant for healthcare workers and policymakers in high endemicity areas; Olatunji et al. showed that the disease burden can be effectively reduced through immunization programs and improvement of cART coverage in children [29]. A retrospective cohort study by Shen et al. reviewed vaccination coverage in Chinese HIV-exposed children, identifying an early infancy diagnosis of HIV and having mothers with better education, as factors associated with highter probability of being vaccinated, while most of the deceased children in the cohort were not vaccinated [6].

Limitations of this study, based on the review of medical records, include the possibility that records may not be complete, considering the wide age range of patients enrolled. However, we included only children with evidence of full vaccination with proper dosing schedules in the determination of immunity to overcome these potential issues. A further limitation of the study comes from the number of patients lost to follow-up because of their referral to infectious disease clinical centers for adults. This hampered the statistical analysis, but also made long-term assessment of the seroprotection rate more difficult.

Also, the small sample size, the wide age range, and the consequent different management in the first years after diagnosis may represent a limitation. A late diagnosis means a risk of more severe immunodeficiency.

A strength of the study was that all patients were followed up at the same referral center, so that antibodies measured in all samples were performed by the same laboratory, which could minimize intra-assay, inter-assay, and inter-laboratory variabilities.

## 5. Conclusions

Despite cART, perinatally HIV-infected children have a lower rate of antibodies against vaccine-preventable disease compared to healthy controls. Although children who received the primary vaccination after the beginning of cART showed a better response to the vaccines, the protective effect of vaccination is reduced over time. This suggests that serological response to the vaccines should be routinely checked in children with HIV infection to test their protection against potentially dangerous infections.

## Figures and Tables

**Table 1:** Population demographic at enrollment.

Characteristic		All 63 (100)	HIV-Uninfected 37 (58.73)	HIV-Infected 26 (41.27)

Sex *n* (%)	Female	34 (53.97)	18 (48.65)	16 (61.54)
	Male	29 (46.03)	19 (51.35)	10 (38.46)
Age, y; median (range)		10.08 (1.68–22.20)	7.09 (2.00–17.38)	13.89 (1.82–22.20)
Country of origin *n* (%)	Italy	55 (87.30)	35 (94.59)	20 (76.92)
	Other, EU	1 (1.59)	0 (0)	1 (3.85)
	Other, non-EU	7 (11.11)	2 (5.41)	5 (19.23)

**Table 2:** Main features of perinatally HIV infected patients at enrollment.

Characteristic	HIV-Infected (26)
HIV disease duration, months; median (range)	18 (1–151)
CD4 cells/mL; median (range)	795 (144–2358)
Patients with HIV RNA <40 copies/mL; *n* (%)	23 (88)
ART; *n* (%)	26 (100)
2NRTI + IP	15 (57.69)
2NRTI + IT	8 (30.77)
2NRTI + 1NNRTI	1 (3.85)
1NNRTI + 1IP	2 (7.69)
Vertical transmission route; *n* (%)	26 (100%)

**Table 3:** Protection rate against measles, mumps, rubella and Hepatitis B in HIV and control at enrollment.

Vaccine			
	HIV-Uninfected 37	HIV-Infected 26	*p*
Measles			
IgG positive %, *n*	83.8 (31)	65 (17)	0.1
Mumps			
IgG positive %, *n*	78.4 (29)	61 (16)	0.1
Rubella			
IgG positive %, *n*	92 (34)	65 (17)	0.02
Hepatitis B			
IgG positive %, *n*	78.4 (29)	45.4 (10) ^1^	0.02

^1^ Antibodies determination against hepatitis B at enrollment was available for 22 patients.

## Data Availability

The data presented in this study are available on request from the corresponding author.
